# Controllable quantum dynamics of inhomogeneous nitrogen-vacancy center ensembles coupled to superconducting resonators

**DOI:** 10.1038/srep33271

**Published:** 2016-09-15

**Authors:** Wan-lu Song, Wan-li Yang, Zhang-qi Yin, Chang-yong Chen, Mang Feng

**Affiliations:** 1 State Key Laboratory of Magnetic Resonance and Atomic and Molecular Physics, Wuhan Institute of Physics and Mathematics, Chinese Academy of Sciences, Wuhan 430071, China; 2 University of the Chinese Academy of Sciences, Beijing 100049, China; 3 The Center for Quantum Information, Institute for Interdisciplinary Information Sciences, Tsinghua University, Beijing 100084, P. R. China; 4 Department of Physics, Shaoguan University, Shaoguan, Guangdong 512005, China

## Abstract

We explore controllable quantum dynamics of a hybrid system, which consists of an array of mutually coupled superconducting resonators (SRs) with each containing a nitrogen-vacancy center spin ensemble (NVE) in the presence of inhomogeneous broadening. We focus on a three-site model, which compared with the two-site case, shows more complicated and richer dynamical behavior, and displays a series of damped oscillations under various experimental situations, reflecting the intricate balance and competition between the NVE-SR collective coupling and the adjacent-site photon hopping. Particularly, we find that the inhomogeneous broadening of the spin ensemble can suppress the population transfer between the SR and the local NVE. In this context, although the inhomogeneous broadening of the spin ensemble diminishes entanglement among the NVEs, optimal entanglement, characterized by averaging the lower bound of concurrence, could be achieved through accurately adjusting the tunable parameters.

Significant progress has been made recently in the field of quantum information processing (QIP) based on hybrid systems, especially for composite systems consisting of solid-state spin systems (e.g., nitrogen-vacancy center ensembles (NVEs), nitrogen substitution *P*1 center ensembles or *Y*_2_*SiO*_5_ spin ensembles) and superconducting resonators (SRs)^1^,^2^,^3^,^4^,^5^,^6^,^7^ or superconducting qubits^8^,^9^,^10^,^11^, which provide a promising platform to study fundamental quantum information science^12^,^13^,^14^,^15^,^16^ and intriguing quantum optical phenomenon^17^,^18^,^19^,^20^. The negative charged nitrogen-vacancy (NV^−^) centers in diamond^21^,^22^,^23^,^24^,^25^,^26^,^27^,^28^,^29^,^30^,^31^ feature excellent coherence properties (e.g., long coherence time in a wide temperature range even at room temperature), and have the ability to coherently couple to various external optical/microwave fields simultaneously^32^,^33^,^34^,^35^,^36^. More importantly, in contrast to the conventional methods relying on electric-dipole couplings, the collective magnetic coupling mechanism for manipulating spin ensembles owns the advantages of weak dissipation and strong coupling. These novel merits ensure the NVEs being one of the ideal candidates for integration into a hybrid quantum system which gathers the strength of each physical system and mitigates the individual weaknesses^8^,^12^,^37^,^38^.

To date, successive experiments have demonstrated strong magnetic couplings between SR and NVE^1^,^2^,^3^,^4^ (or *P*1 center ensemble^5^), and between superconducting gap-tunable flux qubit^39^,^40^ and NVE^8^,^9^, for which, many researchers have paid much attention on the potential applications^41^,^42^,^43^ and also been motivated to make theoretical efforts on intriguing quantum behaviors^19^,^20^, continuous variable entanglement^44^, and quantum simulation on the condensed matter physics^17^,^18^ as well as many-body physics^45^,^46^ in such systems.

However, the inhomogeneous broadening of frequencies regarding the spin particles, caused by the magnetic dipolar interactions with the nuclear or the excess electron spins in diamond, plays a central role of restricting the performance in quantum information storage and transfer^47^,^48^,^49^,^50^, and also induces some complications during the evolution of the system. Hence, a deeper research is highly expected for understanding how serious the inhomogeneous broadening affects quantum dynamics and entanglement generation of multiple NVEs. We emphasize that the multi-NVE dynamics itself is very complicated and could exhibit richer dynamical behavior than the two-NVE case by comparing a three-NVE model with a two-NVE one. Then we show how dephasing induced by the inhomogeneous broadening influences quantum dynamics and entanglement generation in a three-NVE case, where we concentrate on a specific frequency distribution of the inhomogeneous NVE. In our study, we find that the dynamics displays a series of oscillations under various experimental situations, which reflects the intricate competition and balance between the NVE-SR collective coupling and the adjacent-site photon hopping. Influenced by the dissipation of SR and NVE, the oscillations show a slight trend of damping. However, for the dephasing effect caused by the inhomogeneous broadening, a counter-intuitive effect is the suppression of the population transfer between the SR and the local NVE, namely, the dephasing effect enlarges the oscillation periods. In this context, the optimal entanglement among the NVEs could be achieved through accurately adjusting the tunable parameters, such as the Rabi frequency due to the external driving field as well as the hopping rates.

On the other hand, preparation of a high-degree entanglement among three or more NVEs is challenging both experimentally and theoretically. A promising way to overcome this obstacle might be offered by recent development of circuit QED technique in 1D (2D) superconducting resonator array^51^,^52^,^53^,^54^, providing more attractive possibilities to realize distributed entanglement with tunable interactions among multiple separated spin ensembles. Our detailed analysis finds a way to extract proper experimental parameters for the optimal entanglement among the NVEs using existing experimental technologies, even in the presence of large inhomogeneous broadening of the spin ensemble. As such, the present system provides a platform to generate multipartite quantum entanglement of the NVEs embedded in distant sites. In this sense such studies not only provide information of how entanglement evolves with time but also suggest ways toward practical purposes because the entanglement can be controlled by adjusting the tunable parameters^55^,^56^. Therefore, it is desirable to investigate quantum dynamics of the NVEs in different sites, and develop efficient methods for controlling the entanglement dynamics of several distant NVEs.

## Results

### System and model

**Figure 1:**
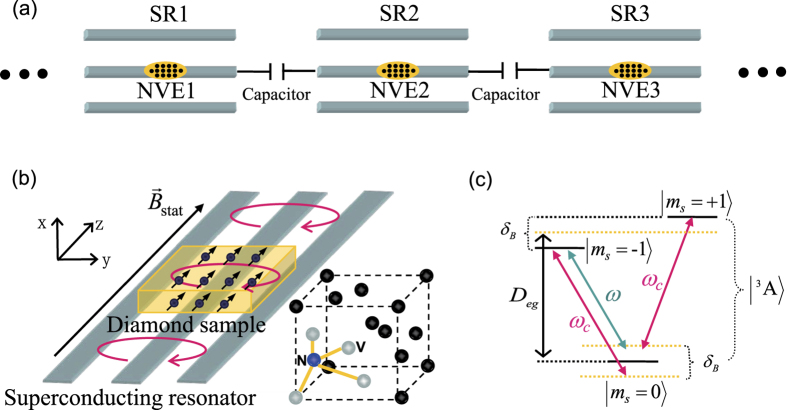
(**a**) The hybrid system under consideration is a circuit QED array consisting of *N* distant NVEs coupled respectively to *N* separate SRs connected by (*N* − 1) capacitors allowing for photon hopping between interconnected nodes. (**b**) A NVE is placed on a resonator’s surface, where a static bias field 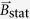 along the *z*-axis is applied to lift the degeneracy of the levels *m*_*s*_ = ±1 of the ground state 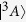. (**c**) Energy level diagram for the NVE, where an additional classical microwave driving field with frequency *ω* is applied to induce the transition 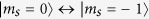 with the detuning −*δ*_*B*_/2 and the Rabi frequency Ω. The quantized microwave field mode of the resonator with frequency *ω*_*c*_ also couples to the transitions 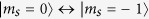 and 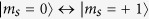 with the detunings 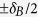.

As illustrated in Fig. 1, the system under consideration is a circuit QED array consisting of *N* distant NVEs coupled to *N* separate SRs respectively. The energy level configuration of the NV^−^ center is shown in Fig. 1(c). Two electrons of the vacancy make a quasi covalent bond with the lone pair of nitrogen atom, and an extra electron located at the vacancy site forms a spin *S* = 1 pair with the residual electron of vacancy. The zero-field splitting *D* of the triplet ground state 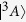 is around 2*π* × 2.88 GHz. With all NV^−^ centers initially prepared in the state 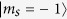 and based on the Raman transition scheme as well as the adiabatical elimination method^57^ (see details in Method), we can obtain an effective Hamiltonian of each site in the subspace spanned by 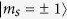 with the following form (in units of 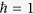)


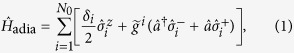


where *N*_0_ is the number of the NV^−^ centers in the spin ensemble, 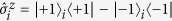 and 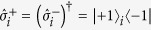 are the Pauli operators for the *i*-th NV^−^ center. 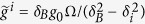 denotes the effective coupling strength between the *i*-th NV^−^ center and SR, where *g*_0_ is the coupling strength of a NV^−^ center to the resonator, Ω is the Rabi frequency of the external driving field. As shown in Fig. 1(b), there exist four crystalline orientations for each NV^−^ center^14^, and all the angles of the possible four orientations of the NV^−^ center with respect to external field 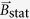 could be identical if the magnetic field is applied along a special direction [100]. In this case, the Zeeman splitting between the states 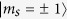 is 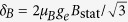 with *μ*_*B*_ the Bohr magneton, 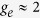 the Lande factor for electron spin. *δ*_*i*_ is the random offset from the central frequency *δ*_*B*_ for the *i*-th NV^−^ center, describing the inhomogeneous broadening of the spin ensemble induced by local strain and interactions with neighboring electronic spins or nuclear spins^58^.

Engineering such a system requires a quantitative understanding of how the inhomogeneous broadening effects on quantum dynamics. Numerically, to fit the inhomogeneous distribution of the frequencies, we adopt the *q*-Gaussian profile function 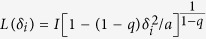 with 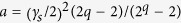, where *γ*_*s*_ is the full width of the frequency at half maximum and the dimensionless parameter 1 < *q* < 3 covers the Gaussian distribution for *q* → 1 and the Lorentzian distribution for *q* = 2 50. Here the key idea in our numerical treatment is to divide the spin ensemble into several subensembles^43^,^45^,^59^ and each one is assumed to be homogeneous, described by


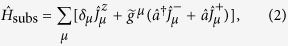


where 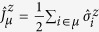 and 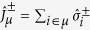 are the collective spin operators. Respectively, *δ*_*μ*_ and 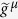 are the random offset and the effective collective coupling strength for the *μ*-th subensemble with *Nμ* NV^−^ centers.

After mapping collective spin-*N*_*μ*_/2 system in 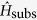 into effective bosonic operators using the Holstein-Primakoff (HP) transformation^60^ 
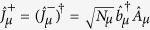
 and 
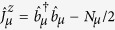
 with 
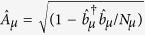
 and 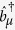 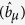 the creation (annihilation) operators, one can obtain following Hamiltonian 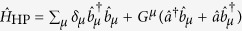 with 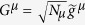 in the case of 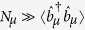, where all the nonlinear items containing 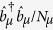 could be safely neglected.

For the whole system, when considering the nonlocal microwave photons hopping between the adjacent sites, we have the total Hamiltonian as


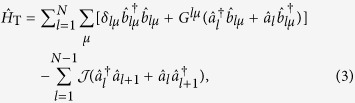


where 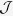 is the hopping rate between neighboring resonators and *l* indicates the *l*-th site. We may assume an uniform distribution of coupling rates across the 1D array, i.e., *G*^*l*,*μ*^ = *G*^*μ*^. The term 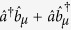 in the Hamiltonian 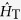 denotes the linear mixing interaction. Actually, for the single-excitation case, the present model is equivalent to the Jaynes–Cummings-Hubbard model, which describes a single photonic mode strongly coupled to a two-level system in each cavity and photons can hop between the cavities. However, in contrast to single atom dynamics, the NVEs have more possibilities of increasing the collective coupling to a point at which counter-rotating-wave interactions become relevant, benefiting from the collective enhancement of the magnetic-dipole coupling. Additionally, the inhomogeneous broadening effect of spin ensemble, which is absent in the single-atom model, attracts even more attention as it may bring some special or novel effects, such as the results presented in the next section.

### Quantum dynamics

As shown below, rich quantum behaviors can be observed using recent experimental technologies even in the presence of large inhomogeneous broadening of spin ensemble. Our system offers a great degree of freedom to control its evolution since the linear mixing process can be controlled by the effective coupling strengths *G*^*μ*^, which are dependent on the Rabi frequencies Ω of the external driving fields. Additionally, the *in-situ* tunability of the circuit elements also offers a channel to control its evolution by modulating the adjacent-site photon-hopping rates 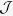. Here we define the collective coupling strength 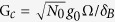 and investigate the single-excitation dynamics of the whole system in the large coupling case 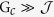, the competition case 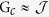, and the large hopping case 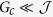, respectively.

The equations describing the dynamics of each boson involved in the system can be written as


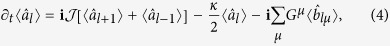



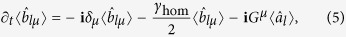


where *κ* is the photon loss rate of the SR, *γ*_hom_ is the dissipative rate of the NVEs, and the dephasing effect is involved by considering the NVE’s inhomogeneous broadening.

**Figure 2 f2:**
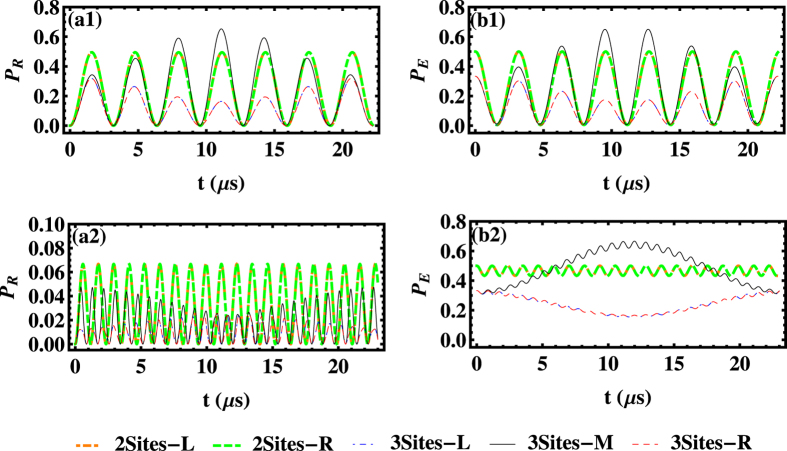
Probabilities of excitation vs time *t* for SRs (a1∼a2) and NVEs (b1∼b2) in the ideal case, where the thin blue dotted-dashed, thin black solid, and thin red dashed lines correspond to the probabilities of excitation in the left, middle and right SRs (NVEs) in the three-site case respectively, and the thick green dashed, thick orange dotted lines represent the probabilities of excitation in the left and right SRs (NVEs) in the two-site case. We employ the parameters *δ*_*B*_/2*π* *=* 80 MHz, 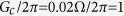 MHz, *κ*/2*π* = 0, *γ*_hom_/2*π* = 0 and 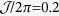 MHz in (a1,b1), 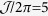 MHz in (a2,b2). Due to symmetry of the dynamics, the thin blue dotted-dashed line and the thin red dashed line are overlapping in each panel.

We first study the ideal case without considering any dissipation/dephasing. In general, multi-NVE dynamics would be more complicated and exhibit richer dynamical behaviors than the two-NVE case. To verify this conjecture, in Fig. 2, we compare the dynamics of a two-site model with a three-site model under the same condition. For example, the initial excitation is seeded in every NVE with equal possibility, i.e., 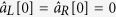, 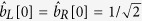 for the two-site model, and 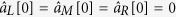, 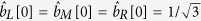 for the three-site model, where *L*, *M* and *R* denote the left, middle and right site respectively. For the two-site model, the dynamics of the NVEs (SRs) in the left site and right site are fully overlapped, oscillating with the amplitude around 0.5 in (a1,b1) and 0.07 in (a2,b2). It implies that the full excitation transfers between the spin ensemble and the local resonator take place simultaneously in both sites due to symmetry. For the three-site model, the middle actor breaks the balance of excitation distribution in the two-site case, presenting an obviously different dynamics from the sideward sites. The total dynamics of the system shows small oscillation periods and large oscillation envelopes with different time-dependent amplitudes. These novel features are absent in the two-site model. Besides, there is no evident difference between the large coupling regime and the large hopping regime in the two-site model. In contrast, the dynamics of these two different coupling regimes can be easily distinguished in the three-site model, exposing more complicated and richer evolution behaviors.

**Figure 3 f3:**
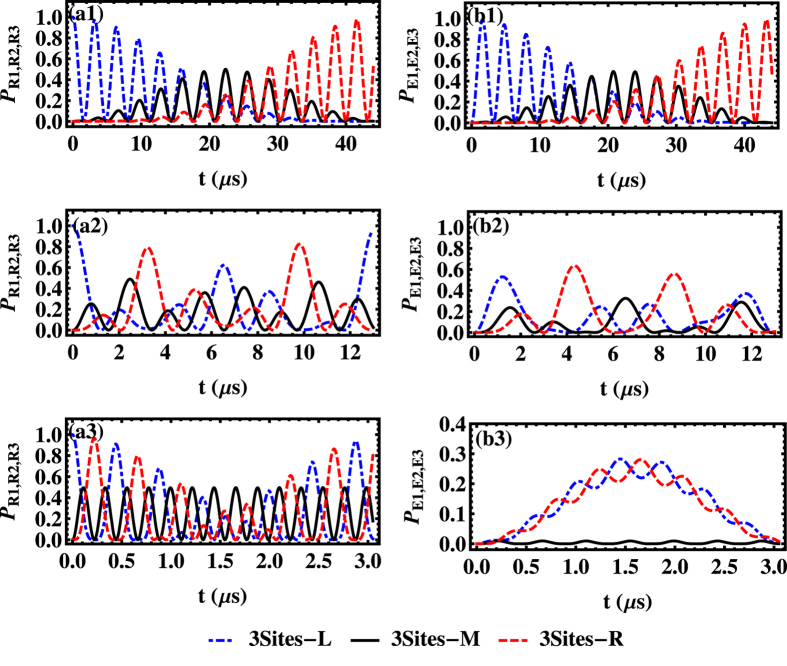
Probabilities of excitation vs time *t* for SRs (a1∼a3) and NVEs (b1∼b3) in the ideal three-site case, where the blue dotted-dashed, black solid, and red dashed lines correspond to the probabilities of excitation of the left, middle and right SRs (NVEs), respectively. We employ the parameter values *δ*_*B*_/2*π* = 80 MHz, *G*_*c*_/2*π* = 0.02Ω/2*π* = 1 MHz, *κ*/2*π* = 0 and *γ*_hom_/2*π* = 0, 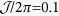 MHz in (a1,b1), 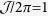 MHz in (a2,b2), 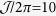 MHz in (a3,b3).

Now we focus on the dynamics of the three-site system in different coupling regimes as shown in Fig. 3. Initially, the excitation is assumed to be seeded in the left SR, i.e., 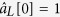, 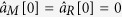, and 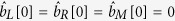. In the large coupling regime 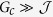, as shown in Fig. 3(a1,b1), the curves representing the population evolution of the NVE in (b1) follow the oscillations of population in the corresponding SR in (a1), implying a rapid and periodic excitation transfer between the SR and NVE at the same site. However, the relatively slow photon-hopping process facilitates a long-period excitation transfer from the left SR to the right one in turn, and so is the reverse. Additionally, the frequency of fast oscillation related to the coupling process is several times as that of the hopping process. Based on the analytical results shown in Method, and using the Fourier transformation, typical oscillation frequencies appear during the dynamical process, as 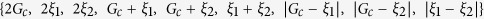. In the large coupling regime 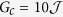, the typical periods could be calculated as around 3 *μs* and 44 *μs*. Due to both the coupling and hopping mechanisms, the features of excitation transfer among the three NVEs are similar to that of excitation transfer among the resonators. Since the central SR simultaneously couples to the side ones through hopping process, the maximal population in the central SR (NVE) is half of that in side resonators (NVEs).

The concrete process can be described in more details: (i) The excitation is initially located in the left SR mode, and then transfers to its corresponding NVE during a period ∼2*π*/*G*_*c*_ due to a strong coupling process; (ii) At the same time, through weak hopping mechanism, the energy gradually flows into the middle site and the right site in sequence during a period 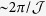; (iii) The JC-type oscillation also takes place at both the middle and the right sites; (iv) The reverse process occurs, which ultimately leads to excitation of the left SR and the corresponding NVE; (v) The above-mentioned process repeats for several times, as shown in Fig. 3(a1,b1).

In the competition case 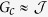, as plotted in Fig. 3(a2,b2), the populations of NVEs and SRs are found to exhibit characteristic oscillations with different frequencies, which is similar to that in the large coupling regime. In the case of 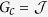, the typical oscillation frequencies involved during the quantum dynamics are extracted as 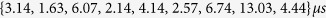. As expected, the situation becomes very abstruse when these two crucial parameters 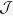 and *G*_*c*_ are comparable.

In the large hopping regime 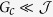, as illustrated in Fig. 3(a3,b3), unlike the behavior in above two regimes, the quantum dynamics exhibits three remarkable characters: (i) The excitation transfer between the adjacent pairs of SRs with fast oscillations dominates the evolution process due to the large hopping rate, and the weak NVE-SR coupling process leads to the excitation transfer between SR and the local NVE with a slow speed. (ii) The populations in the lateral SRs (NVEs) show oscillations between zero and varied maximum, but the population in the middle site oscillates with the fixed amplitudes. (iii) The maximal population in the middle NVE is much smaller than those in the lateral NVEs, namely, the excitation remains mainly in the lateral NVEs. The dynamics of the NVE is a linear superposition of such two oscillations, as shown in Fig. 3(b3), where the ratio between the slow and fast oscillation periods is about 7.5, and a fast and violent oscillation with a weak amplitude is superimposed on the slow oscillation. Here the fast oscillation governed by the photon hopping represents the process of inter-site transfer of excitation, and the slow oscillation governed by the coupling constant *G*_*c*_ is actually a complete transfer of excitation between the nonlocal modes of SR. In the case of 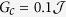, the typical oscillation period times could be calculated as about 0.4 *μ*s and 3 *μ*s.

**Figure 4:**
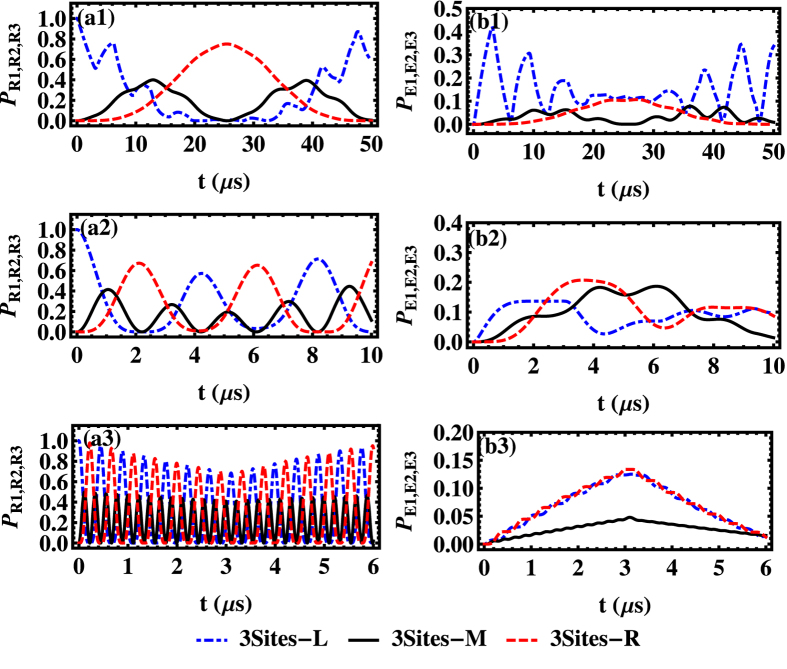
Probabilities of excitation vs time *t* of the three-site model for SRs (a1∼a3) and NVEs (b1∼b3) when both dissipation effect and inhomogeneous broadening are considered, where the blue dotted-dashed, black solid, and red dashed lines correspond to the excitation probabilities of the left, middle and right SRs (NVEs), respectively. The parameter values are as same as Fig. (3) except *γ*_*s*_/2*π* = 20 MHz, *κ*/2*π* = 0.001 MHz and *γ*_hom_/2*π* = 0.001 MHz.

We now turn our attention to the situation involving both the dissipation and the inhomogeneous broadening of the spin ensembles. The dynamical evolution of the populations regarding SRs and NVEs could be numerically simulated by calculating the motion equations for all the bosonic species involved in the dynamics, where each NVE has a *q*-Gaussian distribution with *L*(*δ*_*i*_) around a central spin frequency *δ*_*B*_, and each NVE is divided into several homogeneous subensembles containing *N*_*μ*_ spins. Although *q* determines the specific profile such as how tall the peak of the distribution is and how fast the tails of the distribution fall off, it turns out that different value of *q* will bring similar results about the excitation distribution in each site, through numerical calculation. Therefore, here we only present the results of the Lorentzian distribution with *q* = 2, which is a typical model to study the inhomogeneous broadening of the NV^−^ centers^40^,^43^,^50^. As shown in Fig. 4(a1,b1), the initial excitation is identical to the ideal case and the system displays a series of damped oscillations under various experimental situations, which also reflects the intricate balance and competition between the local NVE-SR couplings and the adjacent-site photon hopping. Physically speaking, the time-dependent superposition of the six harmonic wave functions ultimately determines the time evolution of all the one-excitation states. So the overall dynamics may exhibit some complex interferences or competing effects, and it exhibits an oscillating behavior with the amplitude decaying as time goes by and the enlarged oscillation period. To distinguish these two features brought by the different decoherence channels (the dissipation and the dephasing induced by the inhomogeneous broadening), we have studied their influences separately and found that the amplitude decaying mainly results from the dissipative effects with respect to the NVE and SR, and the presence of inhomogeneous broadening suppresses the population transfer between the SR and the local NVE, namely, the dephasing effect enlarges the oscillation period. From the relation 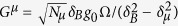, one can find that the values of *G*^*μ*^ decrease with growth of the offset frequency δ_*μ*_, which is related to inhomogeneous broadening of the spin ensemble. That is the reason why the period of the excitation transfer between SR and the local NVE is enlarged, when the dephasing effect is considered.

### Entanglement dynamics

Combined with the microwave photon-hopping process with great flexibility, a fully tunable model for entangling multi-NVEs can be expected in the strong collective-coupling regime, through engineering a quantum control on resonator-assisted Raman transitions with a high degree of freedom. The dynamics of the system shown above also suggests that the high-degree entanglement among the three NVEs could be achieved through accurately adjusting the tunable parameters in the present model.

In our model, both the energy loss of the system and the homogeneous broadening of the ensemble have been considered. With the dissipative and dephasing effects in simulating the entanglement dynamics, an effective approach is the full phenomenological quantum master equation


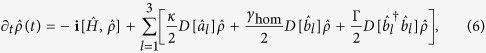


for the Hamiltonian


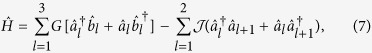


where 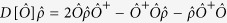, and *κ (γ*_hom_) is the decay rate of SR (NVE). Γ is the dephasing rate of the NVE related to the inhomogeneous width *γ*_*s*_ of the spin ensemble with *γ*_*s*_ = 2Γ under the weak field approximation^50^.

In what follows, we focus on the dynamics of entanglement among three NVEs using the concept of the lower bound of concurrence (LBC)^55^,^56^,^61^,^62^. The concurrence for a general pure tripartite state 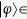 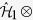 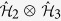 has the following form 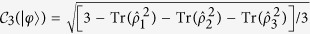
63,64, where 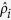 is the reduced density matrix obtained by tracing over the remaining subsystems *j* and *k (i*, *j*, *k* = 1, 2, 3 and *i* ≠ *j* ≠ *k*). This result can be extended to the tripartite mixed state 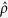 using the convex roof 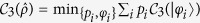
65,66, where 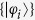 denotes the collection of all the possible pure states into which the mixed state 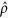 is decomposed as 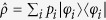 with the positive *p*_*i*_ (normalized already). Here, an exact numerical algorithm is required to find the global minimum in the optimization procedure of 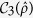 involved in a large number of free parameters, and the lower bound to this measure is available with the following definition^55^,^56^,^61^,^62^,^67^


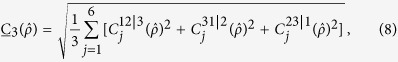



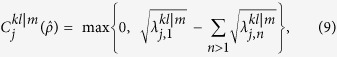


where 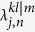 represents the eigenvalues of the matrix 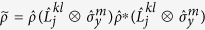 in a decreasing order with 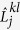 (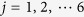) the six generators of the group *SO*(*4*)^68^ acting on the qubits *k*,*l* and 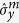 the Pauli matrix acting on the qubit *m*. Note that 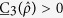 implies an entangled state and a separable state always results in 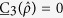.

In our scheme, entanglement of the separate NVEs can be realized through the following channels: (i) Bosonic mode 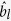 in the *l*-th NVE is entangled with the bosonic mode 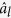 in the *l*-th SR through the local collective coupling. (ii) This entangled state transfers from the *l*st SR to the (*l* ± 1)-th one through the adjacent-site photon hopping with the transfer rate 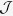, where the transfer process could be mediated by the capacitor acting as a tunable coupler. (iii) Entanglement of the bosonic mode 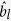 in *l*-th NVE with the bosonic mode 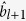 in (*l* + 1)-th NVE is achieved via the linear mixing mechanism. Note that both the collective coupling strength *G*_*c*_ and the hopping rate 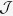 are tunable independently. As a result, entanglement among multiple NVEs in the 1D array of SR can be realized in a controllable way.

**Figure 5:**
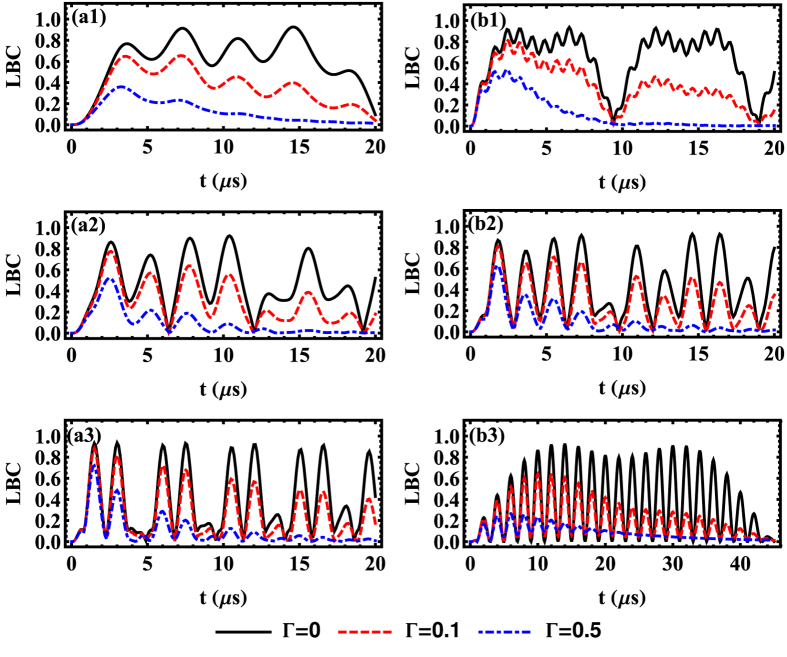
The LBC vs time *t* in the three-site model under the condition of 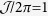 MHz, *G*_*c*_/2*π* = 0.02Ω/2*π *=0.5, 1, 2 MHz, respectively, in (a1,a2,a3) and *G*_*c*_/2*π* = Ω/2*π* =1.6 MHz, 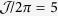, 1, 0.1 MHz, respectively, in (b1,b2,b3). The initial state is *b*_*M*_[0] = 1, and we use Γ/2*π* = 0 MHz (black solid curves), 0.1 MHz (red dashed curves), 0.5 MHz (blue dot-dashed curves), and *δ*_*B*_/2*π* = 80 MHz, *κ*/2*π* = 0.001 MHz, *γ*_hom_/2*π* = 0.001 MHz.

In the presence of both the dissipative and the dephasing effects, Fig. 5 characterizes the time-dependent entanglement of three NVE by the LBC. We find that the inhomogeneous broadening has a detrimental effect on the entanglement of spin ensembles, where the optimal LBC becomes smaller and smaller with growth of the dephasing rate Γ. This implies that, to obtain a high-degree entanglement of NVEs, we have to suppress the inhomogeneous broadening as much as we can using the recent experiment methods, such as the spin echo technology^58^.

Next, two distinct cases are considered. The first case plotted in Fig. 5(a1–a3, [Fig f2], [Fig f3]) is for the fixed value of the hopping rate 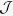 and the increasing collective coupling strength *G*_*c*_. One can find that, for the same hopping rate 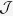, the increase of the driving frequency *G*_*c*_ could induce an obvious growth of oscillation frequency of entanglement and a slight accretion of the maximal amplitude with respect to LBC, in the presence of dephasing effect. It implies that the collective coupling strength *G*_*c*_ controlling the linear mixing interaction process has a positive effect on the entanglement generation among NVEs. The second case presented in Fig. 5(b1–b3, [Fig f2], [Fig f3]), with identical coupling strength *G*_*c*_ and various hopping rates 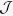, describes a remarkable result that the maximal amplitude of the LBC increases if the hopping rate 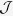 has an increment. Note that this feature is obvious only in the region where the hopping and dephasing rates are small. Additionally, as shown in the small hopping case (Fig. 5(b3)), the endurance period of the entanglement among spin ensembles is extended to be twice of those in Fig. 5(b1), where the hopping rate 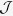 is relatively large. Given the same evolution time, the higher photon hopping rate means more probabilities for photons shuttling between different sites, which results in a larger amount of entanglement among the NVEs located at different positions. Besides, slower photon-hopping rate gives less chances for the dissipation through the SRs in different sites. Thus, it is the reason that the lifetime of the entanglement becomes longer when the value of 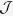 decreases.

**Figure 6:**
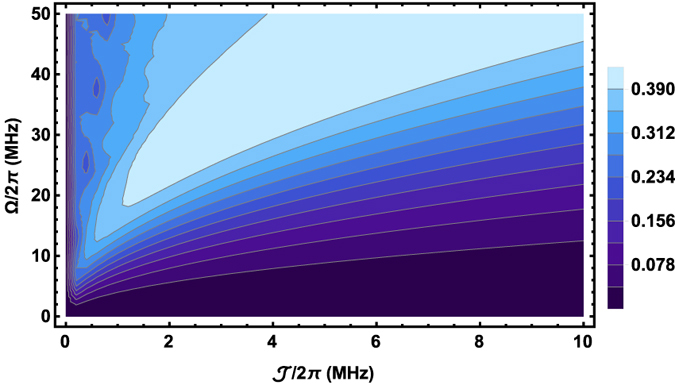
Density plot of the average LBC within the 20 *μs* evolution time in the three-site model vs the hopping rate 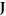 and the driving frequency Ω with the initial state *b*_*M*_[0] = 1, Γ/2*π*  = 0.1 MHz, *κ*/2*π* = 0.001 MHz and *γ*_hom_/ *π* = 0.001 MHz.

Therefore, a rich entanglement dynamics of the NVEs also reflects the intricate balance and competition between the two different kinds of interaction processes. One is the collective magnetic coupling process between the resonator mode and collective mode of the local NVE, and the other is the photon hopping process between the adjacent sites along the whole 1D array. These two related tunable parameters (Ω and 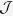) influence the LBC dynamics of the NVEs in different ways such as the maximal amplitude and the duration of the oscillation. To provide a more complete picture about how to achieve an optimized entanglement, the dependence of the average LBC during a certain period of time on the parameter space {Ω, 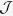} is plotted in Fig. 6, where the optimal point can be extracted. The average LBC reaches the maximal value 0.43 at the point with Ω/2*π* = 50 MHz and 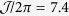 MHz. Around the optimal point, a high-degree entanglement of the spin ensemble can still be obtained. It implies that the LBC evolution can be optimized by appropriately tailoring those key parameters. This provides a useful and effective way to controlling the dynamics of entanglement among the long-distance NVEs distributed in different sites.

## Discussion

We now survey the relevant experimental parameters. Firstly, the external static magnetic field 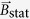 could be applied by placing a copper box containing the resonator and diamonds in the center of two pairs of perpendicular Helmholtz coils^2^,^69^. If we set 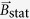 to be 15.4 mT, then the Zeeman splitting becomes *δ*_*B*_/2*π* ≈ 500 MHz. For the inhomogeneous broadening, we choose the frequency *δ*_*i*_/2*π* ranging from −70 MHz to 70 MHz, which ensures both the convergence property and the adiabatic elimination condition 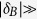 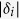. Besides, the values of Ω/2*π* are restricted within the regime {0,100} MHz in our model, which satisfy the adiabatic elimination condition, such as 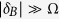, and are available experimentally^70^. Also, the photon-hopping rate 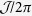 can be tuned within the regime {0,10} MHz depending on the size of the capacitor^53^,^54^,^71^. For a SR with a quality factor *Q* ∼ 3 × 10^6^, the photon loss rate can be calculated as *κ*/2*π* ≈ 0.001 MHz 2. On the other hand, the electron spin relaxation time *T*_1_ of the NVE can reach up to 10 s at low temperature under an appropriately chosen magnetic field^2^,^72^,^73^. The dephasing time reaches *T*_2_ > 600 *μs* for a lower nitrogen density sample with natural abundance of ^13^C at room temperature^58^.

In our model, we have assumed the homogeneous couplings all over the sites of the 1D array, such as the identical number of NV^−^ centers in each spin ensemble, and the identical hopping strength at each pair of SRs. Needless to say, this assumption would not be well satisfied in realistic experiments due to small deviation of the key parameter values induced by the fabrication errors and manipulation inaccuracies. However, treating those imperfections goes beyond the scope of the present paper. Noticeably, the effects of disorder in arrays of coupled cavities have been studied for both small- and large-scale arrays^74^,^75^. We emphasize that the initial excitation can be seeded in the SR or the NVE of any site. Due to symmetry, there are analogical dynamics of the system for the initial excitation in the left NVE or the right NVE. Note that the distinct advantages of the present system, such as the individual accessibility and high tunability of the parameters, make this NVE-SR array an ideal platform for investigating controllable quantum dynamics and other applications in quantum information processing, such as the excitation transfer and entanglement generation between distant NVEs in different sites, which are prerequisites for realization of spin-based distributed quantum networks.

In conclusion, we have considered a hybrid system consisting of multiple NVEs coupled to an array of SRs respectively, and the adjacent SRs are connected by capacitors. We have quantitatively simulated the controlled evolution by modulating some key external parameters, and found the way to extract proper experimental parameters for optimal entanglement of the NVEs using existing experimental technologies, even in the presence of large inhomogeneous broadening of the spin ensemble. Although what we have presented above is for a three-site model, the method and results can be easily extended to a longer array in one dimension. Therefore, our scheme provides another route towards building a distributed architecture, and our findings demonstrate that this hybrid quantum system provides a realistic platform for investigating quantum dynamics of spin ensembles and generating entanglement among distant spin ensembles.

## Method

### Derivation of Eq. (1)

For the circuit QED lattice shown in Fig. 1, the Hamiltonian of each site can be described by (in units of 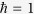)


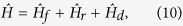



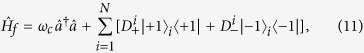



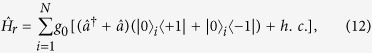



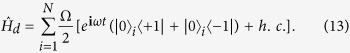



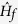
 describes the free Hamiltonian of each site, 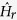 is the NVE-SR interaction term, and 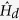 represents the interaction between the NVE and the external classical microwave driving field. *ω*_*c*_ ≈ *D* and *ω* ≈ *D* − *δ*_*B*_ are the resonant frequency of the SR and the driving frequency of the external microwave field. 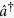
(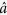) is the creation (annihilation) operator of the resonator mode, and 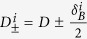 with 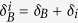.

Using the rotating-wave approximation on 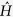 with respect to the free Hamiltonian 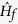, one can obtain a time-dependent Hamiltonian,


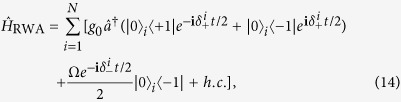


with 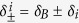. The Hamiltonian 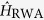 implies that effective transitions between the states 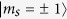 are implemented by Raman coupling processes. Under the condition 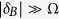, 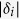 and the assumption that all NV^−^ centers are initially prepared in the state 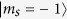, the state 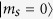 can be adiabatically eliminated by the time-average method in ref. 57, and the effective Hamiltonian in the subspace spanned by 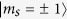 can be deduced as


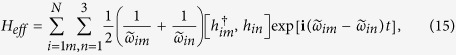



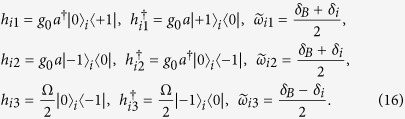


Substituting Eq. (16) into Eq. (15), a simple form of the effective Hamiltonian is available according to the commutation relations. Redefining the Pauli operators 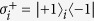, 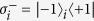 and the coupling strength 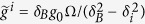, and neglecting the A.C. Stark shift, the effective Hamiltonian is simplified as


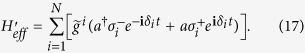


In this way, the two opposite interactions 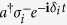 and 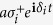 between the SR and NV^−^ center hold the same oscillation frequency. So it is convenient for us to map the effective Hamiltonian into the rotating frame with respect to 
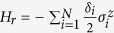
, to get rid of the time factors. Finally, we obtain the simplest and effective Hamiltonian for the system as follows


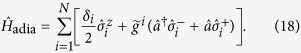


### Analytical solutions to the simplified model

Here we present the analytical solutions of the ideal model, where both the dissipative and the dephasing effects are not considered, and the motion equations can be reduced to


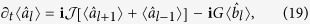



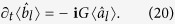


For a given initial state 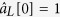, the populations of the SRs (*P*_T1_, *P*_T2_, *P*_T3_) and the NVEs (*P*_E1_, *P*_E2_, *P*_E3_) can be written as


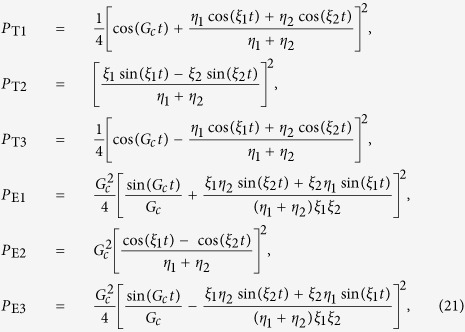


where


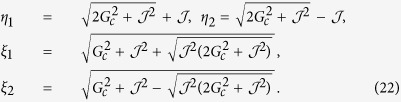


## Additional Information

**How to cite this article**: Song, W.-L. *et al.* Controllable quantum dynamics of inhomogeneous nitrogen-vacancy center ensembles coupled to superconducting resonators. *Sci. Rep.* **6**, 33271; doi: 10.1038/srep33271 (2016).
